# Microencapsulation of *Clostridium difficile* specific bacteriophages using microfluidic glass capillary devices for colon delivery using pH triggered release

**DOI:** 10.1371/journal.pone.0186239

**Published:** 2017-10-12

**Authors:** Gurinder K. Vinner, Goran T. Vladisavljević, Martha R. J. Clokie, Danish J. Malik

**Affiliations:** 1 Chemical Engineering Department, Loughborough University, Loughborough, United Kingdom; 2 Department of Infection, Immunity and Inflammation, University of Leicester, Leicester, United Kingdom; LAAS-CNRS, FRANCE

## Abstract

The prevalence of pathogenic bacteria acquiring multidrug antibiotic resistance is a global health threat to mankind. This has motivated a renewed interest in developing alternatives to conventional antibiotics including bacteriophages (viruses) as therapeutic agents. The bacterium *Clostridium difficile* causes colon infection and is particularly difficult to treat with existing antibiotics; phage therapy may offer a viable alternative. The punitive environment within the gastrointestinal tract can inactivate orally delivered phages. *C*. *difficile* specific bacteriophage, *myovirus* CDKM9 was encapsulated in a pH responsive polymer (Eudragit® S100 with and without alginate) using a flow focussing glass microcapillary device. Highly monodispersed core-shell microparticles containing phages trapped within the particle core were produced by *in situ* polymer curing using 4-aminobenzoic acid dissolved in the oil phase. The size of the generated microparticles could be precisely controlled in the range 80 μm to 160 μm through design of the microfluidic device geometry and by varying flow rates of the dispersed and continuous phase. In contrast to free ‘naked’ phages, those encapsulated within the microparticles could withstand a 3 h exposure to simulated gastric fluid at pH 2 and then underwent a subsequent pH triggered burst release at pH 7. The significance of our research is in demonstrating that *C*. *difficile* specific phage can be formulated and encapsulated in highly uniform pH responsive microparticles using a microfluidic system. The microparticles were shown to afford significant protection to the encapsulated phage upon prolonged exposure to an acid solution mimicking the human stomach environment. Phage encapsulation and subsequent release kinetics revealed that the microparticles prepared using Eudragit® S100 formulations possess pH responsive characteristics with phage release triggered in an intestinal pH range suitable for therapeutic purposes. The results reported here provide proof-of-concept data supporting the suitability of our approach for colon targeted delivery of phages for therapeutic purposes.

## Introduction

The emergence of antibiotic resistant bacteria is a serious global threat to human health. Common bacterial pathogens have become progressively more resistant to standard antibiotics [1]. The development pipeline for new classes of novel antibiotics is not looking promising [2]. There are increasing calls from government health agencies around the world to explore alternative treatment options [3]. Lytic bacteriophages (phages) are viruses that infect and kill bacteria, and they present a promising approach to targeting bacterial infections in a treatment known as phage therapy [4–7]. Phages have been recommended as good candidates for antibacterial therapy [8] because: (i) they are highly specific with a defined host range and therefore they do not disrupt beneficial natural microbiota in the way broad spectrum antibiotics do [9]; (ii) in the presence of an accessible host, they self-amplify *in situ* thereby increasing their dose at the infection site, which may be advantageous for difficult to access infection sites e.g. bacterial biofilms [10] (this dose amplification is however highly dependent on the concentration of both the phage and actively growing bacteria [11], [12]); (iii) they are generally considered to be nontoxic to animals including humans [13], [14] and have minimal impact on non-target bacteria or body tissues when delivered orally [14]; (iv) they may potentially penetrate and kill bacteria within biofilms through production of depolymerases and other enzymes [15–17]; (v) they could be relatively inexpensive to produce using well understood fermentation and downstream processing technology, however, existing lab based manufacturing processes are poorly optimised for producing phage in large quantities thereby incurring high costs if produced under GMP standards required for therapeutic use [18]; (vi) for many species, they can be isolated from accessible sources and they can be modified (using forced phage evolution techniques, under laboratory conditions) to counter phage resistance or alter their inherent properties, and thus if resistance develops, or if a greater spectrum of activity is required, the phage pipeline can be added to without having to start a whole new programme of activity [19]; (vii) they are capable of infecting bacteria which have developed resistance to antibiotics [5].

The use of phage therapy is a particularly promising alternative to broad spectrum antibiotic treatment [3] for acute enteric infections because typically in such infections intestinal concentration of infecting bacteria is high, the causative agent and strain may be suitably diagnosed using rapid diagnostic tools, and application of phage therapy with a sufficiently high initial phage dose would promote rapid *in situ* phage multiplication and decrease in the host bacterial population [11], [20], [21]. Enteric infections worldwide are typically caused by pathogens such as *Escherichia coli*, *Salmonella spp*, *Vibrio cholera* and *Clostridium difficile* [22]. Any such pathogen is potentially a target for phage therapy however there are major barriers to be overcome in terms of understanding the dynamics of the interaction between phage and bacteria in the gut environment, and in terms of the logistics of delivering a stable defined product to the infection site [14].

*C*. *difficile* is the most common cause of infectious antibiotic-associated diarrhoea [23] that occurs in the developed world [24]. There is a significant cost associated with the treatment of *C*. *difficile* infection (CDI); a single CDI case in the UK costs ~£7,000 (Public Health England 2012) and the annual cost in Europe is ~€3 billion. In the USA, treatment for a single case costs ~$12,800 with the annual additional cost to healthcare providers reaching ~$800 million [24], [25].

The organism *C*. *difficile* is a Gram positive spore forming obligate anaerobic bacterium and the infection it causes, CDI is a toxin-mediated disease of the intestinal tract. The clinical outcomes range from asymptomatic colonisation to mild diarrhoea and in severe cases, inflammatory lesions and formation of pseudomembranes in the colon (pseudomembranous colitis), bowel perforation (toxic megacolon), sepsis, shock and even death [26]. *C*. *difficile* is only susceptible to three marketed antibiotics, Metronidazole, Vancomycin and Fidaxomicin and recurrence rates are high and indicate that spores surviving treatment results in re-infection [27].

A lack of suitable *C*. *difficile* phages has limited research on phage therapy for CDI. A recent study [28] however, showed the potential of phages to treat CDI in a hamster model using combinations of *C*. *difficile* phages (a polyvalent cocktail containing between 2 to 5 phages covering a suitable host range was used; the phages delivered to the hamsters were given as simple phage suspensions and bicarbonate was administered to the animals prior to phage delivered orogastrically to reduce the stomach acidity). The phages used in this study all encoded integrases and thus were not typical of phages developed for therapy. However no strictly virulent phages have been isolated for this pathogen and their demonstrated efficacy in multiple model systems suggests that they have significant potential to reduce the *C*. *difficile* burden in the gut environment. Data presented by Nale et al. [28] showed that phages were effective both *in vitro* and in two versions of a relevant hamster infection model, where use of phages reduced colonisation and increased time of survival in the hamster model. Further work has shown that the same phage combinations can be used to prevent and treat infection in a biofilm and an insect model [17].

When free phages are delivered orally for phage therapy or for modulating microbiota [29], there is likely to be variable but significant losses of phage titre by the time phages reach the intended infection site. Potential reduction in phage titres due to phage inactivation attributed to stomach acidity may in part have been responsible for failure of a recent clinical trial aiming to show reduction in acute bacterial diarrhoea symptoms in children using phage therapy [14]. This is primarily due to the acidic conditions encountered in the stomach, and the presence of bile and digestive enzymes and other proteases in the intestinal tract and stomach [30–32]. Therefore, there is a clear need to protect phages against these adverse gastrointestinal conditions by encapsulating them [31–33], and to control their targeted release at the site of infection e.g. in the colon for CDI. The goal of encapsulation is to create a suitable micro-environment to protect phages from the harsh environmental conditions found in the stomach, and also to protect the phages during processing and storage prior to use [34].

Encapsulation technologies are well established for many pharmaceutical drugs however, for phages such technologies may not be suitable. This is because the techniques suitable for conventional therapeutic compounds often involve the use of organic chemicals that may inactivate phages [35], [36]. To overcome this, recent phage encapsulation strategies have used basic homogeniser and extrusion techniques that appear to be less harmful and they maintain phage viability to some extent [31], [32], [37–39]. The lack of suitable encapsulation techniques that allow the precise control of phage encapsulation has been the motivation behind the present work where we have used microfluidic technology to encapsulate bacteriophages.

Unlike homogeniser and extrusion techniques microfluidic based microencapsulation allows exquisite control over each droplet that allows the control of desirable properties [40] including precise loading of phage per particle and uniformity of the particle size and release kinetics. We suggest that combining microfluidic encapsulation with the possibility of being able to select a wide range of available stimuli responsive polymers will enhance the utility of phage encapsulation for a range of therapeutic applications. As such, the encapsulation strategy taken in this work was to microencapsulate *C*. *difficile* specific phages in a pH responsive polymer.

**Fig 1:**
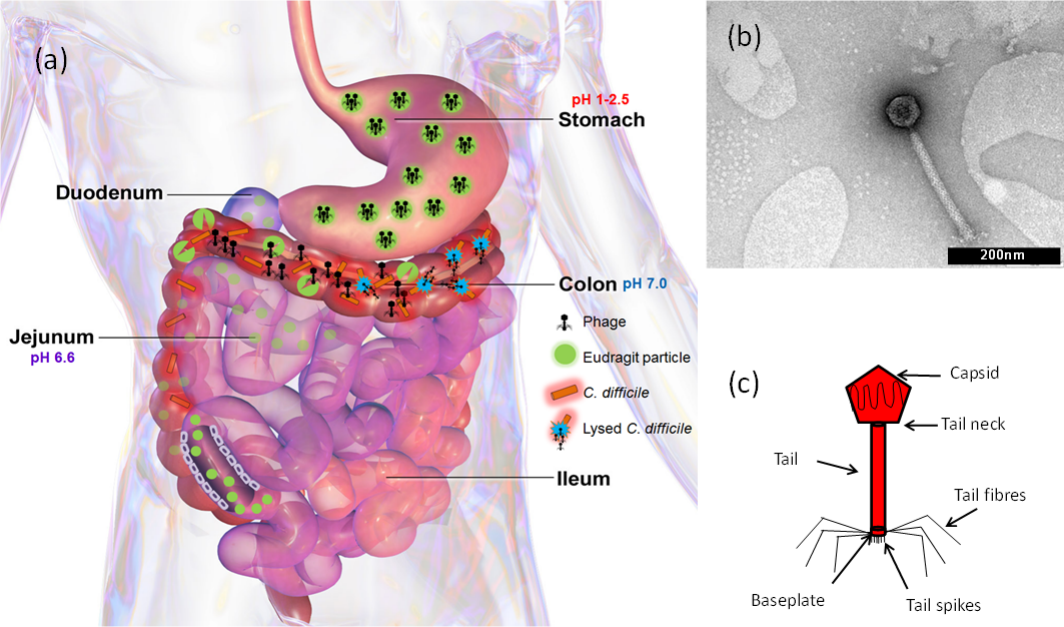
Overview of phage encapsulated in pH responsive microparticles and delivered to the colon. (a) Schematic of the gastrointestinal tract, depicting transit of intact encapsulated phage-loaded microparticles through the stomach and their subsequent disintegration in the colon at pH 7 leading to the controlled release of phages from the microparticles; (b) Transmission Electron Microscopy and (c) schematic of bacteriophage Phi9CD-KM, Myovirus.

**Fig 2:**
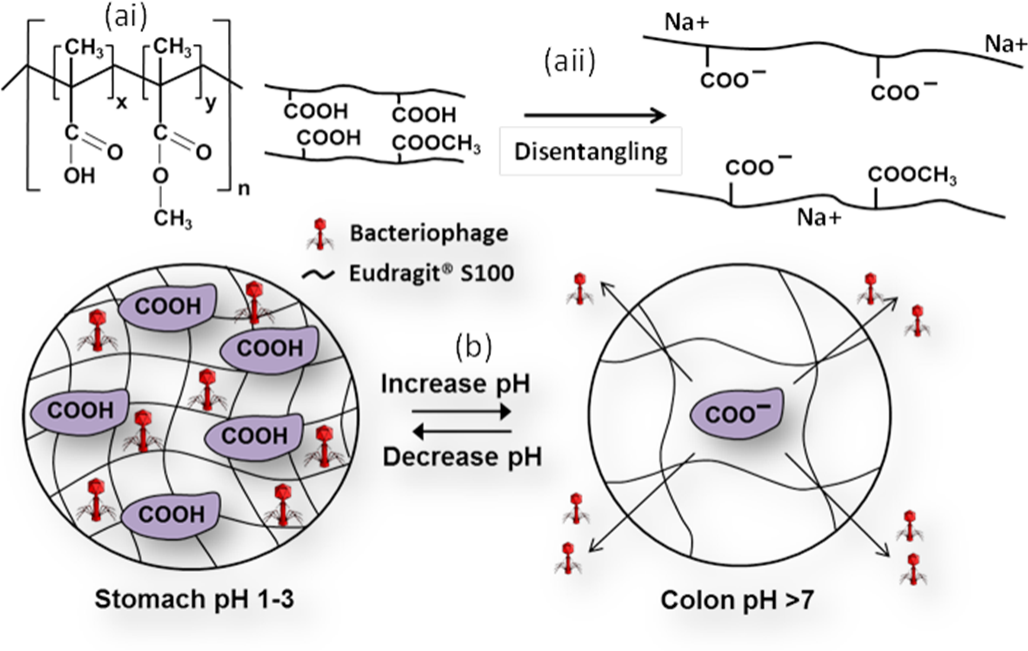
Mechanism of phage release from microparticles. Schematic of ai) the chemical structure of Eudragit® S100, y:x = 2:1 aii) Eudragit® S100 polymer chain separation due to dissociation of weakly acidic functional groups, electrostatic repulsion and polymer swelling; (b) Phage loading/release mechanism from pH responsive Eudragit® S100 microparticles.

The main desired feature of phage microencapsulation presented here is to protect the phages from the stomach acid, and then release them in the infected colon at the typical pH of 7 (Fig 1A). Previously published studies that have used Eudragit® S100 as an enteric coating for colon targeted drug delivery in combination with alginate have shown promise [41]. Alginate is a natural biopolymer which is biocompatible, in other words nontoxic to tissue, biodegradable and relatively cheap. The gelation of alginate may be achieved by addition of high molecular weight polycations such as chitosan, or the addition of low molecular weight polyvalent Ca^2+^ cations [42]. Eudragit® S100 is an anionic copolymer composed of methyl methacrylate and methacrylic acid with monomers arranged randomly on a polymer chain (Fig 2Ai). The presence of weakly acidic carboxyl groups in the polymer chain results in the pH responsive behaviour; in an acidic environment, the groups are uncharged whilst at pH values greater than their pKa (pKa ~ 4), the polymer chains begin to disentangle due to the electrostatic repulsion between the negatively charged carboxyl groups (Fig 2Aii) [43]. The suggested mechanism of phage release is illustrated in Fig 2B. Prolonged exposure to acid will enable the protonation of the carboxyl groups, allowing precipitation of the polymer.

Composite Eudragit® S100 and alginate particles have been used to deliver 5-aminosalicylic acid (5-ASA) and curcumin (both model drugs) to the colon. Duan et al. [44] found that the Eudragit® S100 coating enables resistance to the acidic environment of the stomach, whilst alginate provides muco-adhesive properties which prolong the residence time of the microparticles within the body. Similar investigations carried out by Sookkasem et al. [45] showed the release of curcumin in simulated intestinal fluid at pH 7.4. Pleasingly, as predicted, the curcumin released from the beads was shown to be cytotoxic to the cell line HT-29, demonstrating the efficacy of encapsulation. Vemula et al. [46] investigated the transit of Flurbiprofen to the colon in healthy volunteers through X-ray imaging. The composite tablets released the drug in the colon without disintegrating in the upper GI-tract.

A further novelty of the work reported here is the microencapsulation of phages using microfluidic technology which allows the manufacture of highly uniform micron-sized core-shell particles [47] and using acidified oil for the precipitation of Eudragit® S100. The technique affords exquisite control over the morphology of the microparticles including their size and size distribution, shell thickness and thereby phage release kinetics, phage loading and encapsulation efficiency.

We report here for the first time the use of a glass microcapillary, flow focussing device to encapsulate phages aimed for colon targeted delivery. Microparticles with *C*. *difficile* phage CDKM9 in the core, surrounded by a shell of either Eudragit® S100 or ES100® /alginate matrix were produced using this device. The pH sensitive microparticles were designed to survive the transition of phages delivered orally via the mouth, through the stomach and intestine with phage release in the colon. We demonstrate the ability to manufacture uniform phage entrapped particles for pH responsive site specific delivery.

## Materials and methods

### Chemical reagents

Eudragit® S100 was purchased from Evonik Germany. Miglyol 840, a propylene glycol diester of caprylic/capric acid, was obtained from Sasol Germany and used as carrier oil for the continuous phase. Polyglycerol polyricinoleate (PGPR) soluble surfactant was purchased from Abitek USA and used to stabilise the water/oil interface. 4-amino-benzoic acid, p-toluenesulfonic acid and sodium chloride (Acros Organics, UK) were purchased from Fisher Scientific, UK. The organic acids 4-amino-benzoic acid and p-toluenesulfonic acid were used to trigger *in situ* curing of the droplets in the oil phase. Ethanol and n-hexane were of analytical grade (Fisher Scientific, UK). Ethanol was used to dissolve calcium chloride as the alginate crosslinking agent and n-hexane was used to remove oil from the emulsion respectively. 5(6)-Carboxyfluorescein was purchased from Sigma Aldrich, UK and used to visualize the generated particles. Ultrapure water was obtained from Millipore 185 Milli-Q Plus water filtration system. Sorensen’s buffer 0.2 M was used as a dissolution media for the microparticles and was prepared by mixing sodium phosphate (NaH_2_PO_4_) (for pH 7, 39 ml and for pH 6, ~88 ml) with sodium phosphate dibasic (Na_2_HPO_4_) (for pH 7 6, 1 ml and for pH 6, ~12 ml) (Fisher Scientific UK). Low viscosity alginate was purchased from Sigma Aldrich, UK.

### *C*. *difficile* strain, bacteriophage and transmission electron microscopy

*Clostridium difficile* strain CD105HE1 ribotype 076 was used to propagate the phage as per the method of Nale et al. [28] Bacteriophage CDKM9 is a *myovirus* previously isolated from an environmental water sample as described by Rashid et al. [48]

The identity and purity of the phages used for encapsulation was confirmed using transmission electron microscopy (TEM) analysis. 1 ml of phage stock was centrifuged at 10,000 rpm for 1 hour at 4°C and the pellet was washed two times with 0.1 M ammonium acetate (Fisher, UK) and re-suspended in 100 μl of ammonium acetate. 4 μl of sample suspension was placed on glow discharged pioloform/ carbon coated copper grids which were allowed to stand for 5 min for bacteriophage to bind, 1% w/v uranyl acetate was added to stain the sample. 10μl of deionised water was used to rinse the sample and the prepared sample was left to dry for 24 h. The grids were examined using a JEOL 1220 electron microscope with an accelerating voltage of 80 kV. Digital images were captured using SIS Megaview III digital camera with associated analysis software.

### Culturing *C*. *difficile and* phage propagation

*C*. *difficile*, CD105HE1, was cultured in brain heart infusion (BHI) (Oxoid, Ltd., UK) agar supplemented with 7% defibrinated horse blood (TCS Biosciences, Ltd., UK). Liquid cultures were grown in fastidious anaerobic (FA) broth and BHI broth. *C*. *difficile* was cultured anaerobically in an anaerobic chamber (Ruskinn Technology, UK) using an anaerobic environmental gas mixture composed of 10 vol % H_2_, 10 vol % CO_2_ and 80 vol % N_2_.

Phage CDKM9 was propagated on CD105HE1. Phage stocks were prepared as follows: 0.5 ml of an overnight culture of *C*. *difficile* was grown in FA broth was transferred to 50 ml of pre-reduced BHI broth and incubated in the anaerobic chamber at 37°C. The optical density (OD_550nm_) was measured at hourly intervals until it reached 0.2, after which 100–300 μl (dependent on phage titre of stock) of phage stock was added. The 50 ml tube was incubated overnight in the anaerobic chamber at 37°C for phage amplification to occur. The following day the tube was centrifuged at 2,000g for 10 min at 4°C. The supernatant was filtered through a 0.2 μm filter (Millipore, USA), collected and stored at 4°C until further use.

Plaque assays were carried out using the double layer agar method as described by Mahony et al. [49] and Goh [50]. Briefly, 1.5 ml of 0.4% BHI semi-solid agar and 1.5 ml of salt mixture (0.4 M MgCl_2_ and 0.1 M CaCl_2,_ Oxoid Ltd. UK) were added to 250 μl of overnight *C*. *difficile* FA broth culture and poured onto 1% BHI agar plates and set under a laminar flow hood. 10 μl of phage solution was spotted on the plate (in triplicate), left to dry and incubated in the anaerobic chamber at 37°C overnight. The following day the resulting number of phage plaques was enumerated.

The phage titre was determined by carrying out serial dilutions from the phage collected and each dilution (in triplicate) was spotted on host lawn. Phage titre was determined by counting plaques and expressed as plaque forming units (PFU) per ml.

### Phage sensitivity assay

Phage inactivation (sensitivity) to different experimental parameters used to encapsulate the phages was assessed as follows. The polymer solvents analysed were deionised water (dH_2_O), 0.2M NaCl and BHI broth. The polymer was dissolved in each of these by adjusting the pH to 7.4. Effect of pH on phage was analysed by adding phage to a 0.2 M solution of NaCl with pH adjusted to 2, 3, 4, 5, 6 or 7 by adding 0.1 M HCl or 0.1 M NaOH. To analyse the survival of phage in each parameter 270 μl of each solution was added to a 96 well plate (in triplicate), with BHI broth as positive control, 30 μl of phage stock (10^8^ PFU/ml) was added to each well. At time points 0 h, 6 h and 24 h serial dilutions in BHI broth (270 μl) were performed for each test and spotted (10 μl) on host lawn (prepared as described in the plaque assay section previously). 32 repeats of each dilution were performed for each time point. The spotted plates were dried for 5 min and then incubated in the anaerobic chamber at 37°C overnight. The following day, plaques were counted and PFU/ml calculated.

### Microfluidics experimental set-up

**Fig 3:**
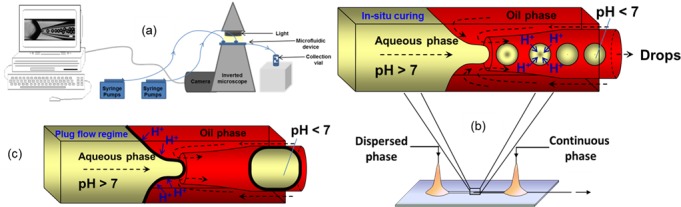
Microfluidic production of microparticles. a) Schematic of laboratory experimental set-up; b) Schematic of droplet production using a glass capillary microfluidic device followed by in situ gelation with 4-aminobenzoic acid dissolved in the oil phase; c) Schematic of the plug flow regime due to premature gelation of Eudragit® S100 with p-toluenesulfonic acid resulting in a high viscosity of the aqueous phase during jet pinch-off. *p*-*Toluenesulfonic acid*, with a pKa of −2.8, is more than 10,000 *times stronger* acid than 4-aminobenzoic acid, with a pKa of 2.38.

The controlled production of microdroplets and microparticles was achieved using in house fabricated glass capillary devices. The experimental setup for drop microfluidic experiments is shown in Fig 3A. Two SGE (1 ml and 5 ml) syringes were mounted on two 11 Elite syringe pumps (Harvard Apparatus, UK) to deliver the aqueous and oil phase to the inner and outer capillary respectively via separate medical tubes. The microfluidic device was positioned on the GXM XD63 optical inverted microscope (GX Microscopes, USA). Formation of droplets started when the dispersed phase reached the orifice of the collection capillary and was observed with a Phantom V9.0 high-speed camera (Vision Research, Ametek, USA) connected to the inverted microscope using x4 magnification lens. The camera was connected to the computer which allowed monitoring and recording of the droplet forming process at 2000 frames per second with a resolution of 576 by 288 pixels. The droplets and particles produced from the device were collected into a glass vial via the collection tube. Table 1 summarises the compositions of the dispersed and continuous phases used for the experimental work reported here.

**Table 1:** Compositions of dispersed and continuous phase.

**Dispersed phase**	D1	0.2M NaCl pH 7.4; 5% (w/v) Eudragit S100;10^7^ PFU/ml
	D2	0.2M NaCl pH 7.4; 5% (w/v) Eudragit S100; 5(6)-Carboxyfluorescein 50μM
	D3	0.2M NaCl pH 7.4; 10% (w/v) Eudragit S100; 2% (w/v) Alginate; 10^7^ PFU/ml
**Continuous phase**	C1	Miglyol 840; 2% (w/v) Polyglycerol polyricinoleate (PGPR); 0.25% (w/v) p-toluenesulfonic acid
	C2	Miglyol 840; 2% (w/v)Polyglycerol polyricinoleate (PGPR); 1% (w/v) p-toluenesulfonic acid
	C3	Miglyol 840; 2% (w/v) Polyglycerol polyricinoleate (PGPR); 1% (w/v) 4-aminobenzoic acid
	C4	Miglyol 840; 2% (w/v) Polyglycerol polyricinoleate (PGPR); 0.75% (w/v) 4-aminobenzoic acid
	C5	Miglyol 840; 2% (w/v) Polyglycerol polyricinoleate (PGPR); 1% (w/v) 4-aminobenzoic acid; ~0.15% (w/v) CaCl_2_; ~3% (v/v) Ethanol

### Microfluidics device fabrication

Experiments were performed using a microfluidic device with counter-current flow focusing (Fig 3B). The device consists of a circular glass capillary tube (Intracel Electrophysiology and Biochemistry Equipment, St Ives, Cambs., UK) inserted into a square glass capillary tube (Atlantic International Technologies Inc., Rockaway, NJ, USA). The inner circular capillary has an internal diameter of 0.58 mm and outer diameter of 1 mm. The outer square capillary has an inner diameter of 1.15 mm and outer diameter of 1.4 mm. 5 cm length of both capillaries was used.

The inner round capillary was pulled using a P-97 Flaming/ Brown micropipette puller (Sutter Instrument Company, Novato, USA) by heating the central region of the capillary approximately 20s until it softens and separates into two parts leaving a sharp tip with a diameter of 20 μm at both ends. The orifice or the nozzle diameter was adjusted to the desired size by abrading the orifice with sandpaper (Black Ice Waterproof T402 Paper, Alpine Abrasives, UK). The orifice sizes used for the experiments were 100 μm and 200 μm. To confirm the inner diameter of the orifice of the inner capillary a Microforge MF-830 microscope (Intracel Electrophysiology and Biochemistry Equipment, St Ives, Cambs., UK) was used. After abrading, the inner capillary was cleaned by blowing compressed air through the lumen and subsequently treated in order to create a hydrophobic inner surface using octadecyltrimethoxysilane (OTMS) (Sigma Aldrich, U.K.).

The square capillary was placed on a square microscope slide (Sigma-Aldrich, UK) and firmly attached with cyanoacrylate glue. The inner round capillary was carefully inserted inside the outer square capillary and axially centred with the help of the microscope. Two syringe needles with plastic hubs (2.5 mm outer diameter and 0.9 mm inner diameter, B-D Precisionglide®, Sigma-Aldrich, UK) were placed at edges of the square capillary and firmly held together with two component epoxy glue (Five Minute ® Epoxy, ITW DEVCON Limited, UK). The aqueous and oil phase was supplied through medical delivery tubes (0.86 mm inner diameter and 1.52 mm outer diameter, Smiths Medical International Limited, UK), attached to syringe needles. The phases entered the square capillary from two opposite ends which were enclosed inside the plastic hubs of the syringe needle. The droplets were collected through a collection tube (1.58 mm inner diameter and 2.1 mm outer diameter, Sigma-Aldrich, UK) which was attached to the inner round capillary (protruding from the plastic hub). The plug flow regime where the formed drop adopts a plug shape and occupies the entire cross section of the collection tube is demonstrated in Fig 3C.

### Preparation of microparticles

The dispersed (aqueous) phase was prepared by dissolving Eudragit® S100 powder (final working concentration of 5% w/v) in either 0.2 M NaCl solution or BHI. 10 M NaOH solution was added dropwise until the solution turned clear and colourless indicating complete dissolution of Eudragit® S100 polymer. To prepare composite particles, 2% (w/v) alginate was dissolved with Eudragit® S100 over heat (~60°C) stirring overnight (formulation D3 in Table 1). For phage encapsulation, the dispersed phase contained 1.6 x 10^7^ PFU/ml of phages in formulation D1 and D3 (Table 1). For dye encapsulation, 10 mM solution of carboxyfluorescein in ethanol was added to 5% w/v Eudragit® S100 dissolved in 0.2 M NaCl to make a final dye concentration of 50 μM (formulation D2 in Table 1).

The continuous (oil) phase was prepared by dissolving 2% (v/v) of PGPR and 1% (w/v) of 4-aminobenzoic acid (formulation C3 in Table 1) or (0.25% or 1%) (w/v) p-toluenesulfonic acid in Miglyol 840. The phases were introduced into the microfluidic device at different flow rates (Table 2), to ensure droplets formed at the entrance of the tapered inner capillary were monodispersed. The flow rates were kept constant throughout each experiment. The droplet formation was followed for periods in excess of 6 h as part to evaluate droplet stability over long periods of operation. The droplet size was analysed using the Image J program (the National Institute of Health, Washington, U.S.). To evaluate droplet stability over long periods of operation, the size of 20 droplets at each time point was measured. The particles were collected in an empty glass vial. Air bubbles are a common issue in microfluidic droplet generation. Air bubbles were avoided by using high-quality gas-tight glass syringes instead of plastic syringes.

**Table 2:** Summary of samples prepared using different microfluidic experimental conditions.

Experiments	Orifice size / μm	Dispersed phase flow rate, Q_d_ / ml h^-1^	Continuous phase flow rate, Q_c_ / ml h^-1^
F1	200	1.2	7.0
F2	200	0.5	3.5
F3	200	0.5	3.0
F4	200	0.5	2.0
F5	100	0.09	2.0

Eudragit® S100/Alginate composite microparticles were prepared as per the procedure above. The dispersed phase composition was 2% (w/v) alginate and 10% (w/v) Eudragit® S100 (formulation D3 in Table 1). Formulation C5 was used for the continuous phase (Table 1). A 5% w/v calcium chloride (Fisher Scientific, UK) solution in ethanol (reagent grade, Sigma Aldrich UK) was prepared and subsequently added to the Miglyol to make-up the appropriate composition of the continuous phase used for alginate crosslinking. The microparticles were left to crosslink overnight at room temperature.

### Washing of microparticles

After collection, the sedimented microparticles were processed by pipetting off excess Miglyol oil. Residual oil was removed by rinsing the particles three times with n-hexane and gently centrifuging at 10 g for 3 min at 4°C. Residual hexane solvent was removed by vacuum drying the particles for 15 min at room temperature.

### SEM imaging of microparticles

20 μl of washed microparticles prepared as outlined above, were pipetted and placed on 13 mm filter disks (0.2 μm pore size, Millipore Ltd. UK). The filter disks with the samples were placed in a -20°C freezer for 24 h. The frozen particles were then placed in a glass freezer dryer jar and connected to a freeze dryer machine at 1 bar pressure and -50°C. The particles were freeze dried for 24 h to allow removal of any residual liquid. The particles were viewed using a benchtop Scanning Electron Microscope (Hitachi TM3030 Table top Microscope).

### Confocal microscopy

Carboxyfluorescein encapsulated microparticles were viewed using a Confocal Laser Scanning Microscope (CLSM) (BioRad Radiance 2000 MP with a Nikon Eclipse TE300 inverted microscope).

### Dissolution of microparticles

2% (w/v) of washed particles were suspended in prewarmed 10 ml simulated intestinal fluid, 10 mg/ml pancreatin in 0.05mM KH_2_PO_4_ at pH 6 or 7 (Sigma Aldrich, UK) under agitation conditions at 37°C. For acid exposure 2% (w/v) of washed particles were suspended in prewarmed 10 ml simulated gastric fluid, 3.2 mg/ml pepsin in 0.2 M NaCl at pH 2 (Sigma Aldrich, UK) for an exposure period of 3 h (reported mean gastric emptying times in human subjects is typically less than 3 hours for both pre-fed and fasted states [51]), under agitation conditions at 37°C. Particles were subsequently centrifuged at 2,000 g for 15 min and re-suspended in simulated intestinal fluid (pH 7) under agitation conditions at 37°C until complete dissolution of particles. For enumeration of phage released, plaque assays were used (as described previously) by removing 10 μl of sample at hourly intervals, serially diluting in BHI broth and spotting on a host bacterial lawn. The PFU/ml was determined the following day at each time point.

### Statistical analysis of results

Comparison of sample means using 2 sample t-tests and evaluation of 95% confidence intervals for data was carried out using the statistical software Minitab 18.

## Results

### Production of phage encapsulated core-shell microparticles

Highly monodispersed core-shell Eudragit® S100 droplets were produced under optimised hydrodynamic conditions using 0.75% (w/v) 4-aminobenzoic acid to precipitate the polymer by ion exchange (a process termed polymer curing). Stable production of uniform droplets required optimisation of the hydrodynamic conditions. Microfluidic devices can be controlled *in situ* by direct microscopic observation. Hydrodynamic conditions were optimised in real-time by adjusting fluid flow rates during each experiment until uniform droplets were produced. Three different droplet generation regimes were observed depending on the relative magnitude of the shear, inertial and interfacial tension forces acting during droplet formation. These forces were modulated by changing the formulation of the phases introduced into the device and the flow rates of the continuous and the dispersed phases.

**Fig 4:**
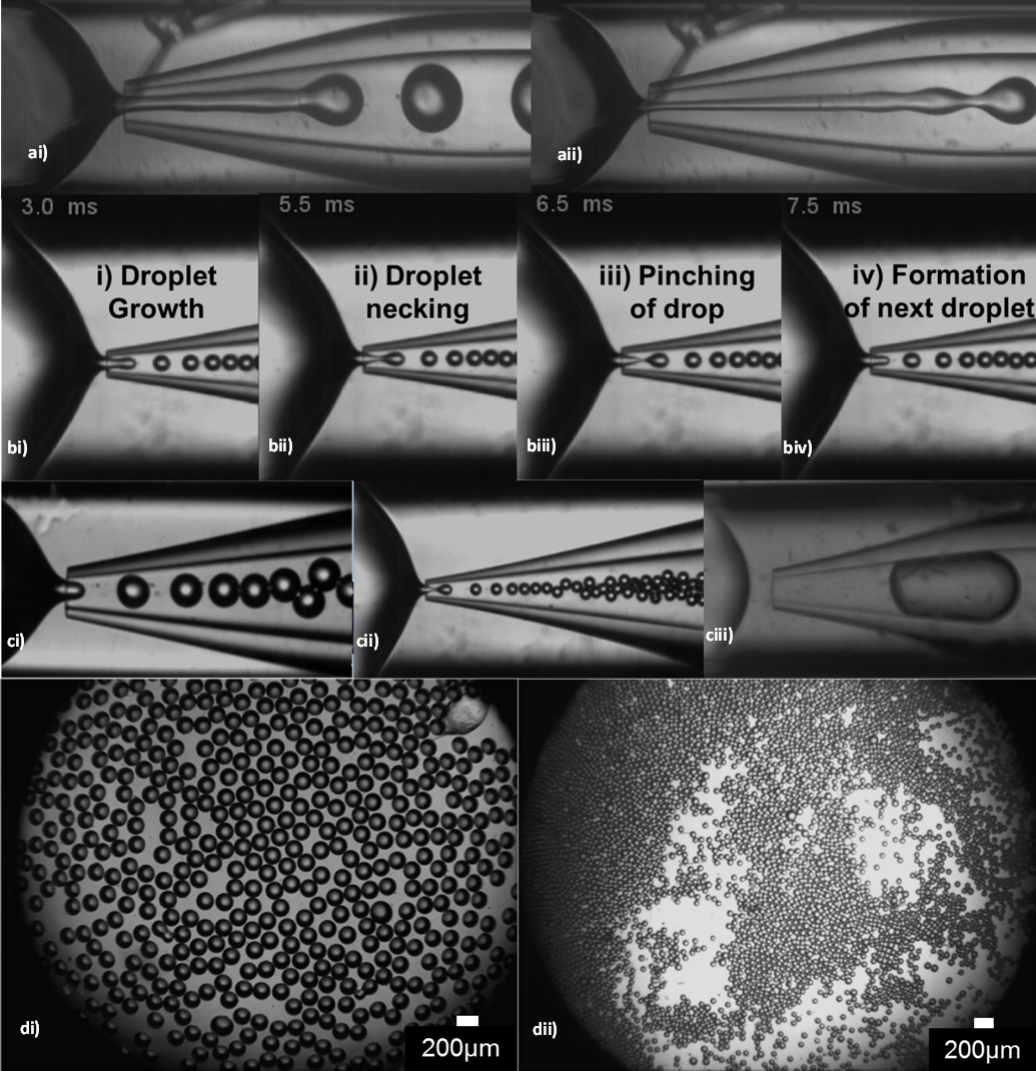
Production of W/O emulsions composed of 5% (w/v) Eudragit® S100 dispersed in Miglyol 840 with 2% (w/v) PGPR and 0.75% (w/v) 4-aminobenzoic acid. ai) Widening jetting at Q_c_ 3 ml/hr and Q_d_ 0.9 ml/hr. The jet length is approximately 9 times the orifice diameter and the drop diameter is 400 μm; aii) Narrowing jetting Q_c_ 6 ml/hr and Q_d_ 0.5 ml/hr. The jet length is approximately 11 times the orifice diameter and the drop diameter is 280μm; b (i-iv) Stages of droplet formation in the dripping regime; ci) The dripping regime during experiment F3 (Table 2); cii) The dripping regime during experiment F5 (Table 2); ciii) The plug-flow regime at the orifice diameter of 200 μm, Q_c_ = 0.06 ml/hr and Q_d_ = 0.3 ml/hr. The curing was achieved using 1% (w/v) p-toluenesulfonic acid. The average drop diameter is ~700 μm; di) Droplets produced in (ci), average droplet size is 135 μm; dii) Droplets produced in (cii), average droplet size is 80 μm.

One regime which was found to be unsuitable for stable microparticle formation was ‘jetting’. ‘Jetting’ is characterised by the long jet created inside the collection capillary away from the orifice resulting in unstable breakup of the droplets at various positions along the jet. Jet widening (Fig 4Ai) occurred due to the high velocity of the dispersed phase compared to the continuous phase causing the velocity of the dispersed phase in the collection capillary to decrease until eventually the jet breaks into large drops due to the Rayleigh-Plateau instability. The transition from ‘dripping’ to ‘widening jetting’ occurs at the critical Weber number of the dispersed phase. The Weber number is a measure of the relative magnitude of inertial forces compared to the interfacial tension forces, $We_{d}=\rho_{d}d_{jet}V_{d}^{2}/\gamma$, where *ρ*_*d*_ is the density of the dispersed phase, *d*_*jet*_ is the jet diameter, *V*_*d*_ is the mean velocity of the dispersed phase and *γ* is the interfacial tension. In Fig 4Aii, the inner phase is stretched into a longer and narrower jet due to the higher flow rate ratio, *Q*_*c*_/*Q*_*d*_ compared to that in Fig 4Ai, but the drops are still formed in ‘widening jetting’ regime. Alternatively, when the velocity of the outer phase in the collection capillary is much larger than the velocity of the inner phase, a narrowing jet is formed producing relatively small droplets. The transition from ‘dripping’ to ‘narrowing jetting’ occurs at the critical Capillary number [52], but this transition was not observed in this study. In either case, the droplet breakup remained unstable due to the imbalance of forces brought on by the high flow rate; either due to the inner or the outer phase. The viscosity of the phases also plays a significant role (shown later).

To enable better control of the droplet generation process and in order to reliably produce monodispersed droplets, the ‘dripping regime’ worked best. Here, the droplets were formed closer to the orifice of the collection capillary (Fig 4B) because the interfacial tension forces dominated over the inertia of the dispersed phase and the viscous stress forces from the continuous phase. Droplet formation began as the dispersed phase entered into the tapered collection capillary (Fig 4Bi). The dispersed phase did not come into contact with the capillary wall due to repulsion from the hydrophobic surface of the treated capillary wall which resisted wetting by the aqueous phase. The continuous phase forced the dispersed phase to elongate axially (Fig 4Bii), collapsing the dispersed phase and producing a neck behind the forming droplet. As the elongation progressed further, the neck thinned into a thread (Fig 4Biii), eventually breaking the dispersed phase and releasing the droplet (mother plug). Following retraction, the process restarted with the dispersed phase protruding at the orifice of the collection capillary (Fig 4Biv). In this dripping regime, the formation of one droplet took 4.5 ms, thus 222 droplets were formed each second and subsequent droplets were produced at the same position as the previous droplets [53], [54]. The balance between the drag force and interfacial tension and negligible shear in the collection capillary after drop formation resulted in uniform droplets. The dripping regime occurs when the Weber number of the dispersed phase and the Capillary number of the continuous phase are both below their critical values. The Capillary number (*Ca*) represents the ratio between the viscous forces and interfacial tension across an interface between the two phases [52], [53], [55]. The Capillary number of the continuous phase is given by: *Ca*_*c*_ = *μ*_*c*_*V*_*c*_/*γ*, where *μ*_*c*_ is the dynamic viscosity of the continuous phase, and *V*_*c*_ is the mean velocity of the continuous phase. For experiments conducted using the dispersed phase flow rate of 0.5 ml h^-1^ and the continuous phase flow rate of 3 ml h^-1^, Fig 4Ci, *Ca* was below 0.1 leading to a dripping regime [53]. The viscosity of the dispersed phase depended on the concentration of Eudragit® S100 and alginate in the different formulations investigated and varied between 5–50 mPas.

Changing the droplet size is an important feature of microfluidics enabling control over phage loading and particle size. Droplet generation in the dripping regime was optimised over a range of flow rates thereby enabling control of the droplet size whilst maintaining a high degree of the size uniformity. This was done in real-time whilst observing (using the microscope camera) the change in the resulting droplet size. When increasing the flow rate of the inner phase relative to the outer phase, the size of the droplets increased; this trend was well documented and observed in many experimental and numerical studies [55]. Typically, the following relationship exists between the drop diameter, *d*_*drop*_, the orifice diameter, *d*_*orifice*_, and the flow rate ratio [56]: *d*_*drop*_/*d*_*orifice*_ ∝ *(Q*_*c*_/*Q*_*d*_*)*^−*x*^, where *x* is about 0.4. In accordance with this equation, when the flow rate of the outer phase was increased, the size of the droplets decreased. This was due to the increased pressure and shear stress exerted by the outer phase to break-up the inner phase into droplets.

Droplet size was also controlled by changing the orifice size of the collection capillary (Fig 4Ci and 4Cii). The plug flow regime occurred due to the high potency of the acid (p-toluenesulfonic acid) in the oil phase resulting in precipitation of the polymer at the water/oil interface during droplet formation. p*-*toluenesulfonic acid, is a significantly stronger acid (compared with 4-aminobenzoic acid) and resulted in premature polymer curing and a rapid increase in the interfacial tension and the viscosity of the dispersed phase. As a result of this phenomenon droplets/particles produced were bigger than the orifice size (Fig 4Ciii). The dripping regime could not be established, irrespective of the fluid flow rates, and the droplet production rate was very low.

Orifice diameters of 200 μm and 100 μm produced an average droplet size of 135 μm and 80 μm respectively (Fig 4D) for experiments using formulation D1 (Table 1) and microfluidic experimental conditions F3 and F5 (Table 2). In both cases, the overall droplet size was smaller than the diameter of the orifice minimising the likelihood of contact between the dispersed phase and the orifice walls and associated wetting. The flow rates were reduced when the orifice diameter was changed from 200 μm to 100 μm, to prevent the transition to the ‘jetting regime’ which would occur due to the high velocity of the two phases entering the collection capillary. Droplets produced by varying the experimental parameters (as described above) resulted in highly uniform droplets with a coefficient of variation of their sizes of less than 2% (Fig 4D).

**Fig 5:**
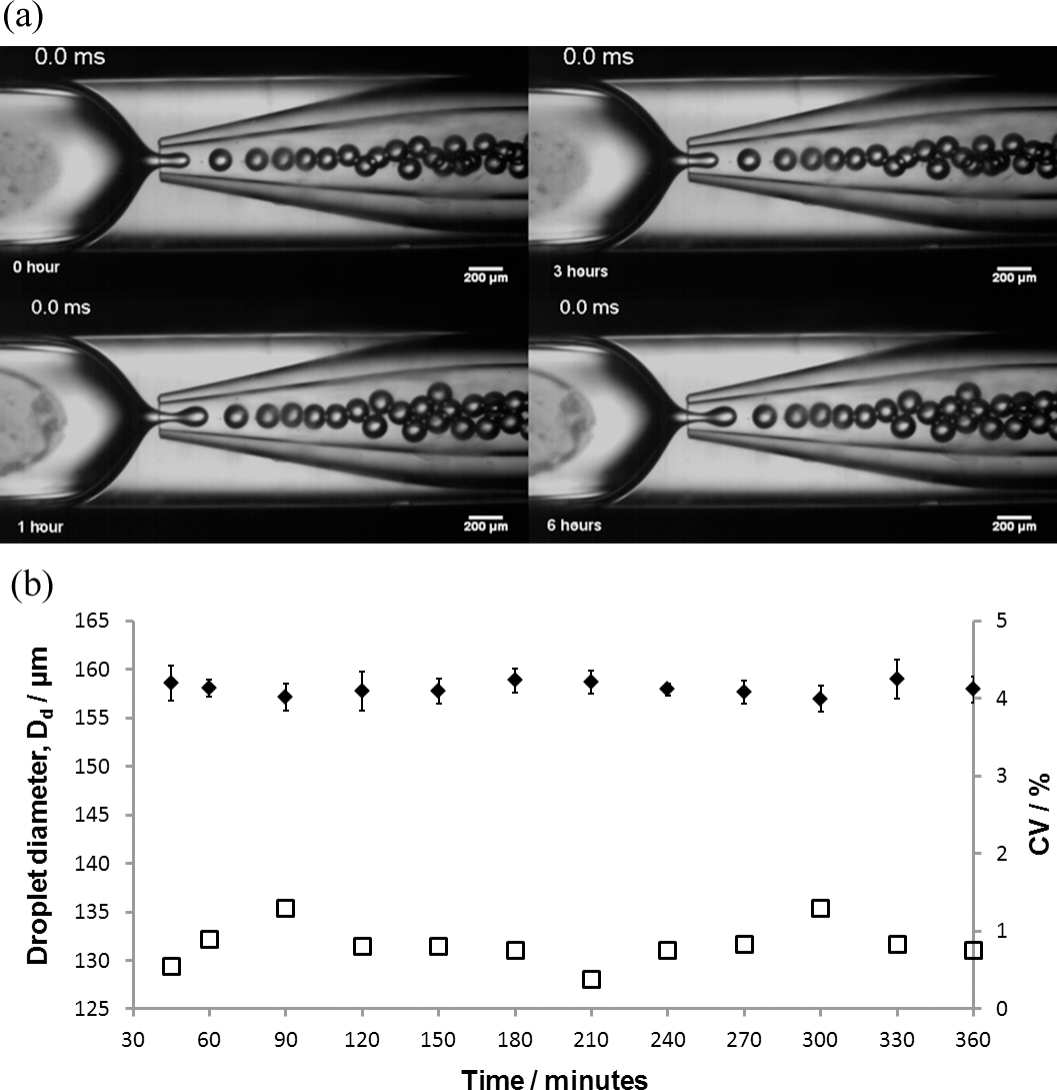
Long term stability study of a glass capillary microfluidic device in generating water-in-oil droplets. **Aqueous phase: 1% (w/v) Eudragit® S100 and oil phase: 2% (w/v) PGPR in Miglyol 840.** (a) Snapshots of droplet generation over six hours (b) Device function over six hours with corresponding droplet size and coefficient of variation (CV) (D_d_ = ◆; CV = ☐). Q_d_ = 1.2 ml/hr, Q_c_ = 7 ml/hr, orifice size = 200 μm). Error bars represent one standard deviation (20 droplets counted per time point).

In order to test the reliability of the device, the microparticle generation experiment was run for 6 h (using experimental conditions F1, Table 2) whilst examining the device integrity and droplet generation behaviour. Visually, the device remained stable over the 6 h period (Fig 5A). There was no wetting of the collection capillary which is usually observed when the aqueous phase comes in contact with the capillary wall resulting in polydispersed drops or even the inversion of the emulsion from a water-in-oil to an oil-in water emulsion [57]. Therefore, pre-treating the collection capillary prior to device setup allowed the aqueous phase to resist coming in contact with the capillary wall. At all times, the dispersed phase remained at the centre of the continuous phase stream due to the 3D geometry of the device and perfect coaxial alignment of the two capillaries.

Throughout the microparticle generation process, no air bubbles were seen in the device which can affect the size of the droplets, by restricting the flow of the dispersed phase. A stable flow pattern was observed without interruption or change in phase behaviour. The device remained functional and identical throughout the six hours of droplet generation; this demonstrated that the device was a reliable means of phage encapsulation contained in the aqueous phase. The stability of the droplet size over the six hour period is shown in Fig 5B. The variation of the droplet size was less than ± 4μm. The coefficient of variation (C.V., a standardized measure of the dispersion of the particle size distribution) measuring the ratio of the standard deviation to the mean droplet size was less than 2%; it is generally accepted that a C.V. value below 3% for a particle size distribution is considered highly monodispersed as demonstrated here; thus the glass capillary device was able to produce highly uniform droplets. All experiments were replicated three times; in each case the flow rate and droplet diameter remained consistent. All post-processing was carried out in the same tube resulting in <1% loss of particles due to processing. The particle sizes were reproducible after all post-processing steps.

### Confocal microscopy of carboxyfluorescein labelled microparticles

**Fig 6:**
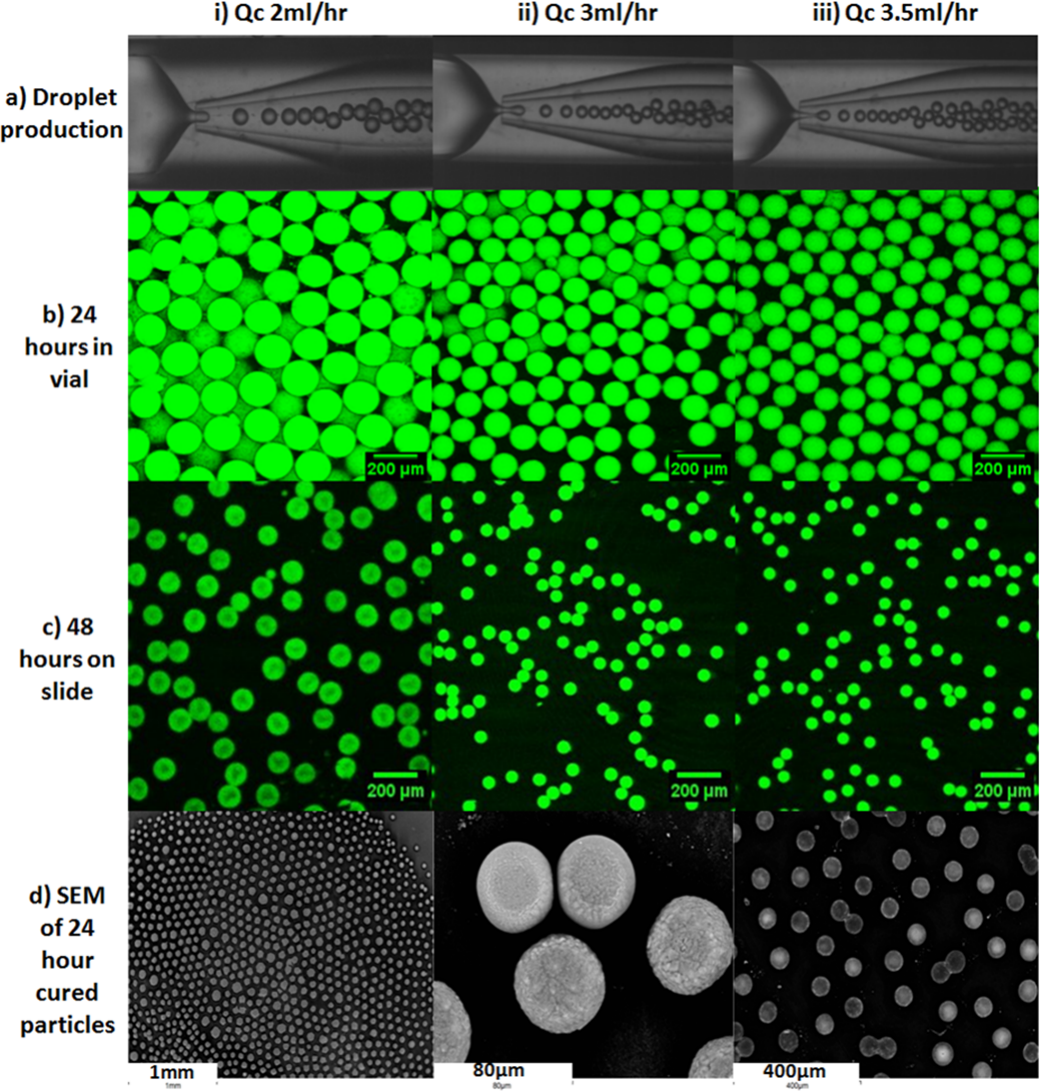
Microscopy images of carboxyfluorescein encapsulated particles cured in acidified oil, dried on a microscope slide. (ai-iii) production of droplets in microfluidic device at different Q_c_ flow rates; (bi-iii) corresponding confocal images of particles cured for 24 hours and 48 hours (ci-iii) produced in (ai-iii); (di-iii) scanning electron microscope images of 24 hours cured particles.

A number of formulations using carboxyfluorescein as the encapsulated agent were evaluated in order to find formulations suitable for encapsulation of phage in microparticles formed using the microfluidic device. Carboxyfluorescein encapsulated particles were generated using optimised flow rates of the inner and outer phase; F2, F3, F4 (Table 2). The average droplet diameter increased with decreasing Q_c_, from 109 μm (F2), to 131 μm (F3) to 170 μm (F4); this was expected due to a decrease in shear and pressure forces acting on the dispersed phase jet with decreasing Q_c_ (Table 2). The change in particle size during curing was observable in real-time using confocal laser scanning microscopy (CLSM) (Fig 6). The encapsulation efficiency of the fluorescent dye was 100%, as no emission was detected in the oil phase.

The influence of curing time on particle size was investigated for microparticles cured using 4-aminobenzoic acid. A significant difference was observed between the mean droplet size in the collection capillary just after formation (discussed above and shown in Fig 6A) and the mean particle size in the collection vial for samples F2 (104 μm), F3 (113 μm), F4 (155 μm). There was no significant difference between the mean particle sizes for samples F2—F4 precipitated for 0 h, 24 h or 48 h. Shrinkage of particles was observed after being left to dry on a glass slide and a similar change in diameter was observed with the SEM analysis, which was due to the loss of water from the particle core and associated shrinkage/collapse of the gel network (Fig 6).

**Fig 7:**
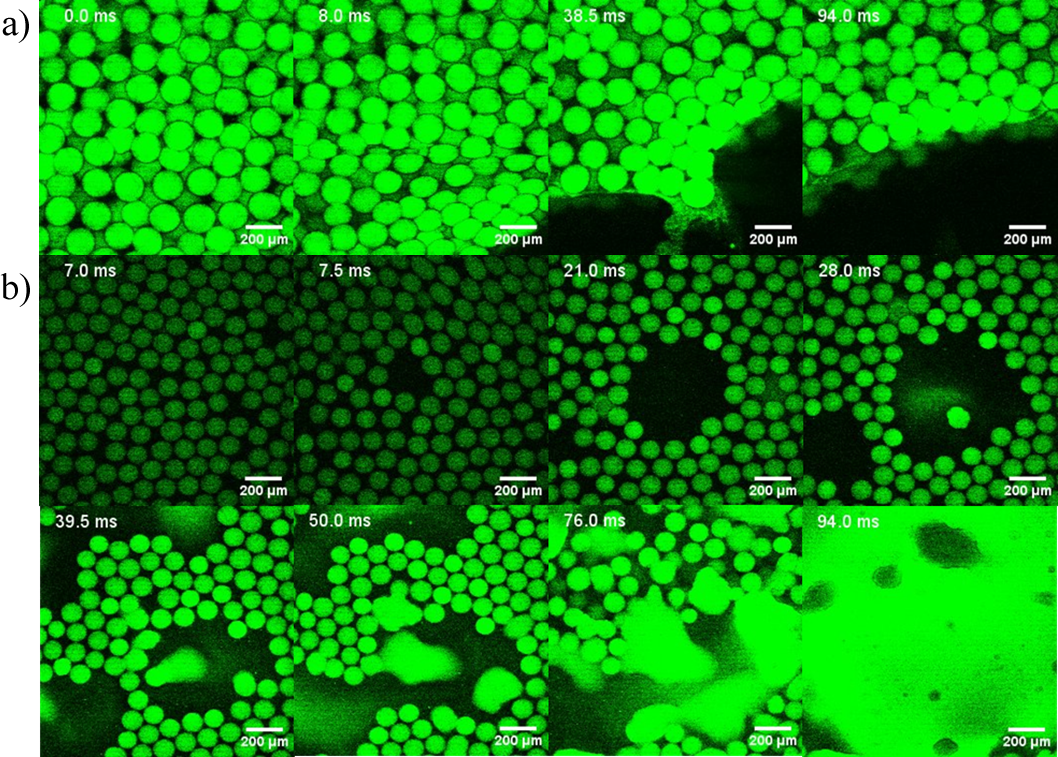
Confocal images showing pH responsive behaviour. Time lapse snapshots (in milliseconds, ms) of carboxyfluorescein (50 μM) encapsulated particles composed of 5% (w/v) Eudragit® S100 (Formulation D2, Table 1). a) exposure of ~170 μm particles to 0.1M HCl; b) dissolution of ~170 μm particles in 0.1M NaOH. Snapshot time series in milliseconds (ms).

To mimic the strong acidic environment encountered during digestion, a 0.1 M hydrochloric acid solution was added to the Eudragit® S100 particles loaded with carboxyfluorescein (a surrogate hydrophilic model drug). The time lapse imaging showed no release of the dye into the surrounding environment (Fig 7A). This suggested that Eudragit® S100 microparticles were capable of retaining the dye and maintaining the integrity of the particles in an acidic environment.

The particles were then subjected to an alkaline environment using a 0.1 M solution of sodium hydroxide, thereby increasing the pH to basic. Leaching of the dye into the surrounding environment was observed and the particles completely lost their structural integrity (Fig 7B). The pH response of the Eudragit® S100 was almost immediate and a burst release of the dye was observed starting from 40 ms. The ionisation of the polymer chains when exposed to sodium hydroxide was immediate with a burst release of the dye as expected due the increased concentration of hydroxyl ions. These results demonstrated the feasibility of encapsulating a water soluble agent in pH responsive microparticles using formulations that allowed microfluidic fabrication of microparticles.

### Transmission electron microscopy

TEM analysis confirmed that the CDKM9 bacteriophage belonged to the *myoviridae* family. Typical features revealed a capsid head of approximately 70 nm attached to a tail and baseplate of approximately 200 nm (Fig 1B). *Myoviridae* phages contain dsDNA in a non-enveloped capsid; their tails are contractile enabling host cell penetration. The six tail pins and fibres are also characteristic of this type of phage (Fig 1B and 1C). The host range analysis for this phage has previously been reported by Rashid et al. [48]

### Effect of formulation parameters on phage survival

**Fig 8:**
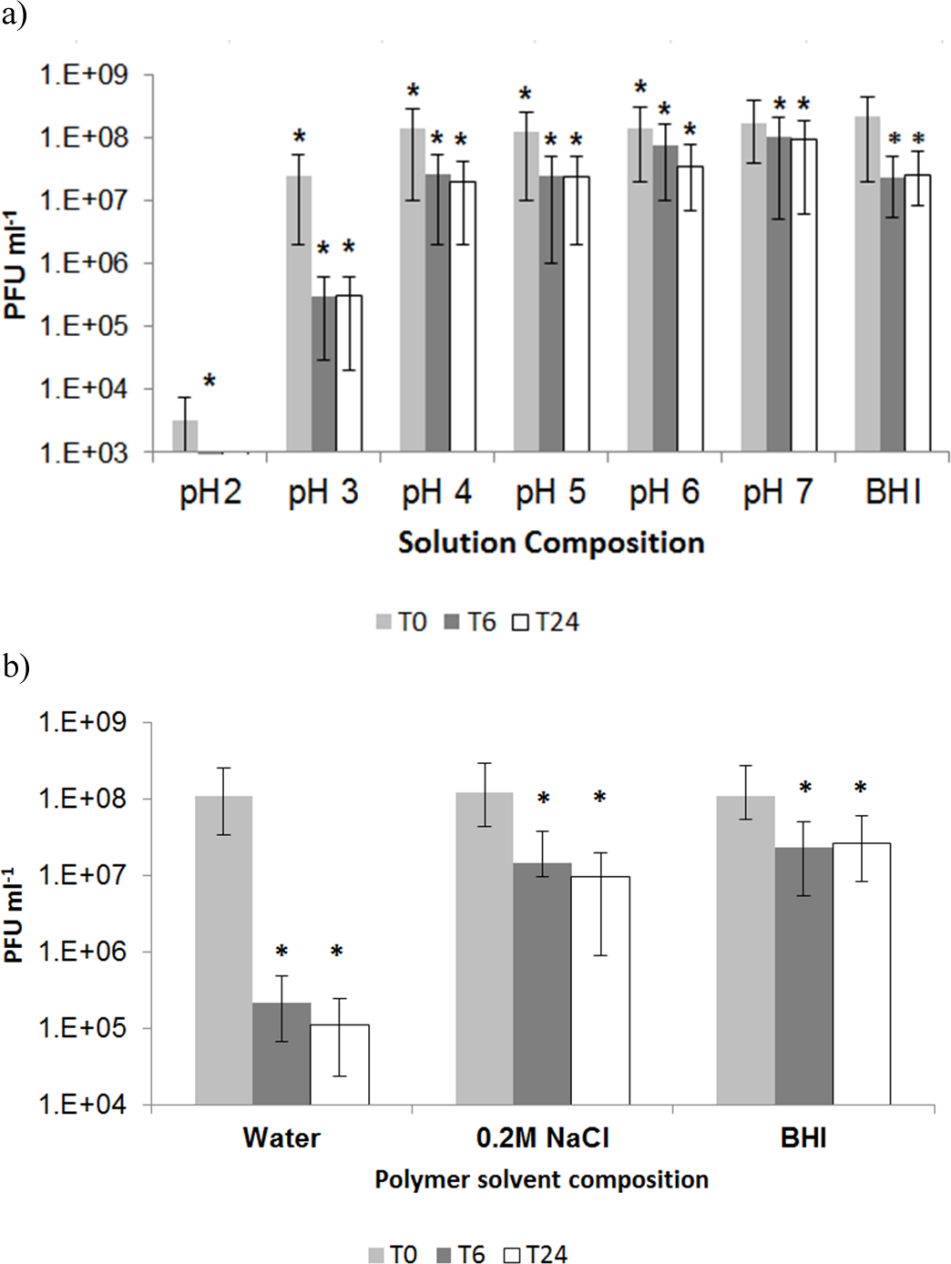
Influence of formulation parameters on free phage viability at various exposure times. a) effect of pH on free phage viability in 0.2 M NaCl solution. * indicates significantly different phage titres using a 2 sample t-test at each condition compared with phage in BHI (controls) at T0 (p < 0.05). Error bars indicate 95% confidence intervals for means.; b) effect of polymer solvent on free phage viability. T0 point denotes time between 0–10 min, T6 exposure to solution for 6h and T24 exposure for 24h. * indicates significantly different phage titres using a 2 sample t-test at each condition compared with phage at T0 for each composition (p < 0.05). Error bars indicate 95% confidence intervals for means.

The effect of different formulation parameters on free phage viability and therefore their impact on phage encapsulation was evaluated. Little work has been done to date on formulation of *C*. *difficile* specific phages showing the effect of physical and chemical environmental factors on phage viability. Free phage in BHI alone were used as a control and compared with phage preparations subjected to different formulations. Firstly, the effect of pH on phage viability was tested for phages that were suspended in a 0.2 M sodium chloride solution at different pH. The phage lost nearly all activity after 10 min of exposure to pH 2 (Fig 8A). Phage viability was significantly improved when the solution pH was above pH 3. Although there was a statistically significant reduction in phage viability for phages exposed to solutions at pH 6 and pH 7 the 95% confidence intervals for the means do overlap suggesting a modest difference in means and not dissimilar to data observed for phages exposed to BHI over a 24 h period (Fig 8A). Phage titres reduced by about one log at pH 4 and pH 5 (Fig 8A).

Phages are usually stored in broth (e.g. BHI broth used here), however, dispersing phage in a polymer solution may adversely impact phage viability. The effect of different dissolution media used to dissolve Eudragit® S100 on phage activity was investigated (Fig 8B). The greatest loss of phage titre was in a polymer solution where the polymer was dissolved in water, and the pH adjusted to pH 7, a 3 log reduction in phage titre was observed. Water was therefore not used for phage formulation.

Eudragit® S100 was also dissolved in 0.2 M sodium chloride solution (Fig 8B) as this has been previously shown to help protect phages from osmotic shock [58]. Phage viability over 24 h of exposure to 0.2 M solution of sodium chloride showed that the phage titre was reduced by ~1 log (Fig 8B). DNA may be lost from phage capsids under high osmotic pressure and therefore the presence of salt is an important consideration for phage stability and receptor binding [58], [59]. Phage viability in 5% (w/v) Eudragit® S100 dissolved in BHI broth was tested showing around ~1 log reduction in phage titre similar to results for 0.2 M sodium chloride.

Phage stocks stored in BHI broth at 4°C showed no greater than ~1 log reduction in phage viability stored over a period of 2 months. Storage temperature between 4°C and 20°C had no significant effect on the phage titre for phages stored in BHI broth (data not shown).

### Phage release in simulated gastric fluid

**Fig 9:**
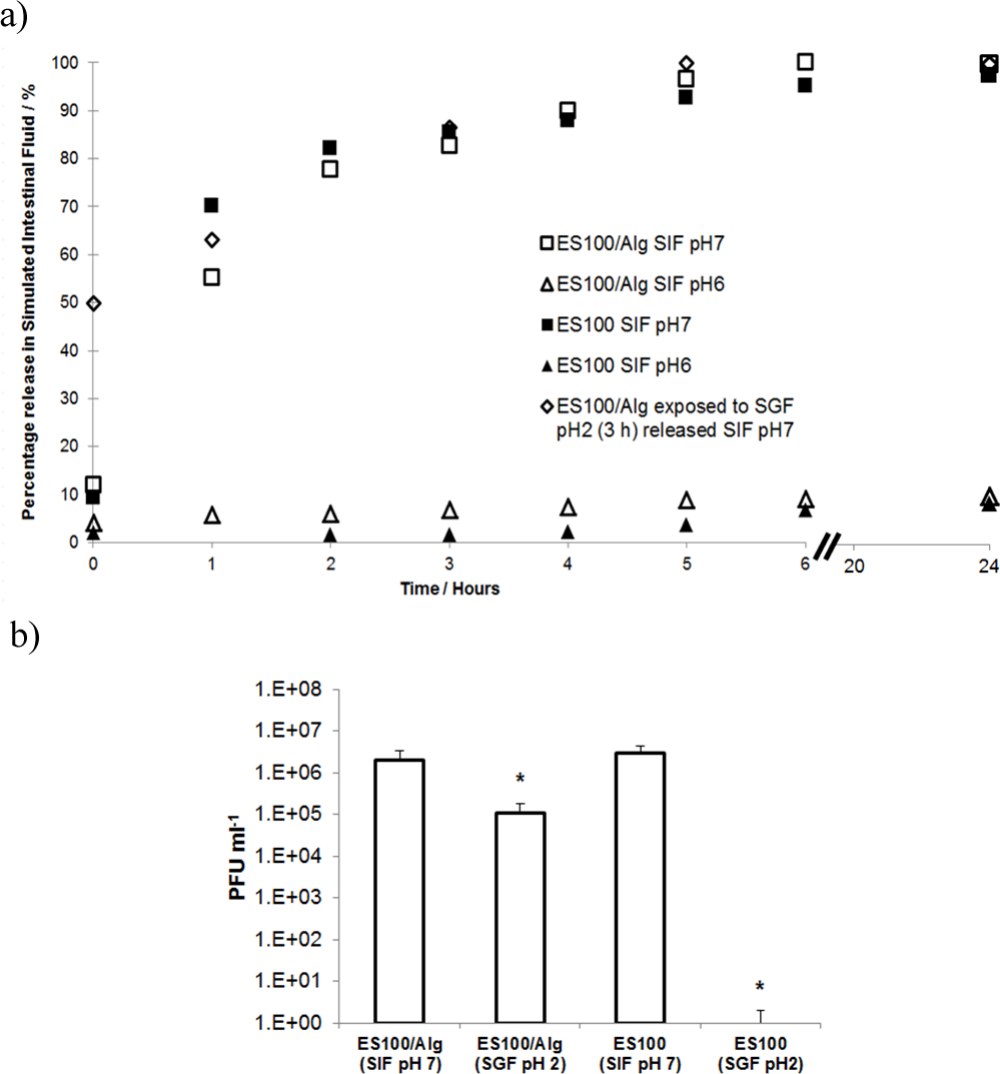
Release of encapsulated phage from microparticles. a) Phage release kinetics from Eudragit® S100 (ES100) and ES100/Alginate microparticles exposed to Simulated Intestinal Fluid (SIF) at pH 6 and pH 7 and from ES100/Alginate microparticles exposed to Simulated Gastric Fluid (SGF, pH 2, 3 h exposure) followed by release in SIF at pH 7; b) Quantitative titres of phages released at 24 h in Fig 9a above. Samples labelled pH 2 were exposed to SGF (pH 2, 3 h exposure) followed by release in SIF at pH 7. * indicates significantly different phage titres (p < 0.05) for a 2-sample t-test of each sample compared with phage release from ES100/Alginate (SIF pH 7). Summary statistics for data are presented in Table 3.

Following washing and removal of Miglyol, encapsulated phage particles were added to shaking solutions containing simulated intestinal fluid. The release of phages in simulated intestinal fluid was assayed over 24 h at two different pH values (Fig 9A). Eudragit® S100 is a pH responsive polymer, known to dissolve at around pH 7. Near 100% (typically > 95%) encapsulation of phages was observed following the entire encapsulation and washing procedures. For pure Eudragit® S100 microparticles, an initial burst release (around 10% of the phage loading) was observed at pH 7 suggesting phages were rapidly able to permeate across the porous microparticle shell. Initially, there is a large phage concentration gradient due to the concentration of phages in the microparticle compared with the outside solution. This gradient drives phages to diffuse into the external solution. The concentration gradient gradually reduces and the phage release slows down over time. Release of phage at pH 7 may be attributed to polymer shell swelling as polymer chains begin to disentangle upon exposure to the buffer and ion exchange occurs between the counter ions H^+^ replaced by Na^+^ and dissociation of the carboxylic acid groups [43]. Phage release in pH 7 was fast (burst release) with most release taking place within an hour of exposure to buffer. Even over a 24 h exposure period to SIF at pH 6 phage only ~ 10% of encapsulated phage was released. 100% release of phage at pH 7 corresponded with a calculated phage dose of ~3.1 x 10^8^ PFU ml^-1^.

**Table 3:** Summary of microencapsulated CDKM9 release results (2-sample t-test for means).

Sample ID	Mean / PFU ml^-1^	StDev / PFU ml^-1^	Lower 95% CI	Upper 95% CI	p-value	Sample size (n)	Null hypothesis
ES100/Alg (SIF)	2.1 x10^6^	4.5 x 10^5^	1.4 x 10^6^	2.8 x 10^6^	-	4	NA
ES100/Alg (SGF)	1.1 x 10^5^	1.8 x 10^4^	7.6 x 10^4^	1.3 x 10^5^	0.000	4	Mean = 2.1 x 10^6^
ES100 (SIF)	3.1 x 10^6^	7.6 x 10^5^	1.3 x 10^6^	5.1 x 10^6^	0.121	3	Mean = 2.1 x 10^6^
ES100 (SGF)	0	NA	NA	NA	NA	4	NA

Notes: Reported mean values are after 24 h exposure to simulated intestinal fluid; CI = confidence interval; p < 0.05 indicates rejection of null hypothesis;

Phage encapsulated in pure alginate microparticles were completely inactivated following acid exposure to simulated gastric fluid (see supplementary information, S1 Fig). With the addition of alginate to Eudragit® S100 particles, significant protection of phage against acidic pH in simulated gastric fluid was achieved (Fig 9B). There was around ~1 log reduction in phage titre in comparison with encapsulated phage not exposed to simulated gastric fluid. The calculated dose for 100% release of phage at pH 7 in SIF following SGF exposure was ~ 1.1 x 10^7^ PFU g^-1^.

Pure Eudragit ES100 microparticles exposed to simulated gastric fluid (pH 2) for 3 hours resulted in complete inactivation of phage with no detected phage release in simulated intestinal fluid (Fig 9B).

### Storage stability of encapsulated phage

**Fig 10:**
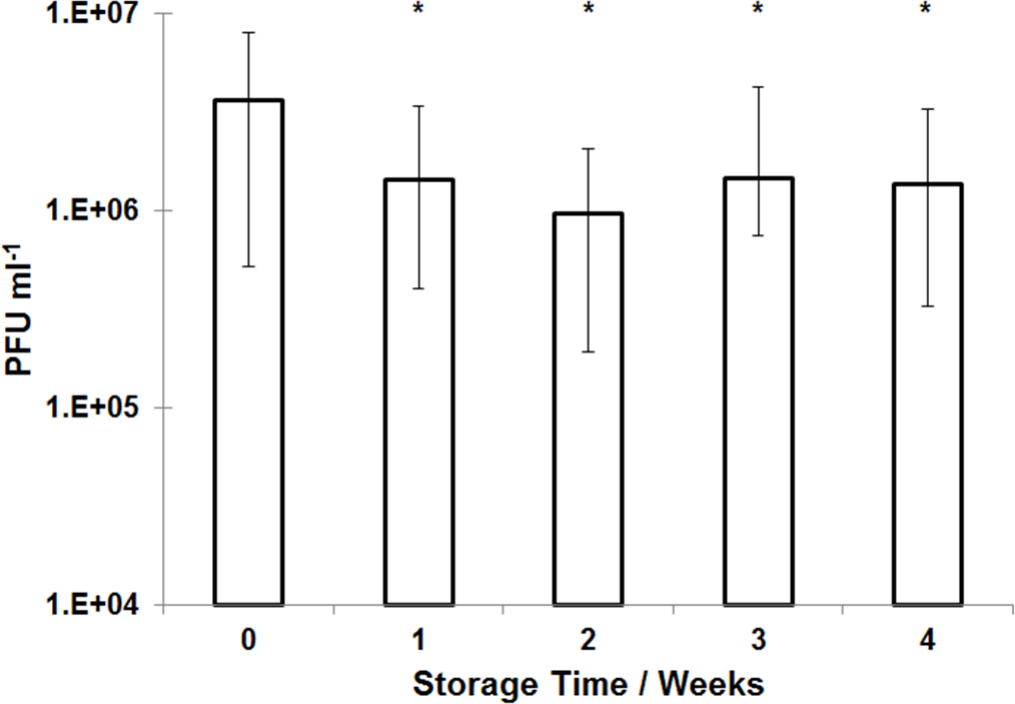
Storage stability of encapsulated CDKM9 phage in ES100/Alginate microparticles refrigerated at 4°C. Phage titre was evaluated by exposing microparticles to simulated intestinal fluid. * indicates significance as compared with sample at week 0 (p < 0.05) using a 2-sample t-test.

ES100/Alg microparticles stored over the course of 4 weeks under refrigerated conditions (at 4°C) showed a modest drop in phage titre (Fig 10). Phage titre dropped from ~ 4 x 10^6^ PFU ml^-1^ (4 x 10^8^ PFU g^-1^ of microparticles) at week 0 to 1 x 10^6^ PFU ml^-1^ (1 x 10^8^ PFU g^-1^ of microparticles) after 1 week of storage. Thereafter, the titre remained stable up to week 4.

## Discussion

A limited range of microencapsulation techniques have previously been employed for bacteriophage encapsulation, with extrusion based droplet formation the most frequently used method [31], [33], [37], [60–64]. None have employed microfluidic fabrication techniques used here. The principle driver for encapsulation has been to protect phages from the stomach acidity. In a significant number of previously published studies, the sizes of phage encapsulated microparticles have tended to be considerably larger than those reported here (an order of magnitude bigger). Little consideration has previously been given to the control of the particle size distribution, the phage loading per particle and the resulting heterogeneity of the release kinetics from each particle [31–33], [37], [61]. A key advantage of the microfluidic fabrication process used here is the precision with which uniform small droplets (mean size ~100 μm) containing encapsulated phage may be prepared under low shear conditions resulting in near 100% phage encapsulation efficiency.

The importance of establishing a stable dripping regime means that each droplet was produced at the same position; it may therefore be assumed that the disruptive forces of the same magnitude acted on each individual droplet and these forces became negligible after pinch-off. This is a rare occurrence because in conventional bulk emulsification systems, the droplets are continuously being subjected to shear which typically varies widely across the mixing container or valve [65], [66]; this is true when encapsulation is carried out using conventional homogenisation or atomisation techniques [67–69]. Continuous excessive exposure to shear results in the droplets breaking further into smaller droplets resulting in emulsions with a high polydispersity index [70]. The microfluidic fabrication process provides a highly controlled uniform shear environment resulting in monodispersed microparticles.

It is well known that phage are sensitive to physical and chemical stresses including shear [71], [72], temperature [72], pH [73], ionic strength [74], exposure to organic solvents [35], [36] etc. Formulations used for phage encapsulation require careful selection of constituents and encapsulation conditions. Phage CDKM9 was shown to be highly sensitive to acidic pH. Similar results have been reported previously for a number of other therapeutic phages including a *Salmonella* phage Felix O1 (a myovirus) [31], a *Staphylococcus aureus* bacteriophage K (also a myovirus) [33], [37] and for an *E*. *coli* specific phage exposed for 5 min at pH 2 [33]. This loss of phage activity highlights the need to protect the phage from the harsh acidic environment of the stomach if controlled doses of phage are to be reliably delivered at the infected colon. Our results show that phage CDKM9 may be acceptably formulated in a low molarity salt solution containing mixtures of dissolved polymers alginate and Eudragit. The observation that pH conditions typically found in the colon do not detrimentally affect phage activity is promising as viable phages need to be released at the site of infection in the colon (where the environmental pH may vary from neutral to alkaline) and remain viable after release. Previously McConnell et al. [75] reported that the pH of the colon can fall during bacterial infection to around or just below pH 7; in such a case, the released phage should remain viable and capable of lysing the infecting *C*. *difficile* bacteria causing CDI. Osmotic damage to phage during phage processing and storage is an important consideration [76]. Governal and Gerba [77] reported activity of an *E*. *coli* phage dropped by 2 log upon exposure to ultrapure water. Our results also suggest that phage CDKM9 titre drops upon exposure to deionised water. Phages from the *Myoviridae* family, are known to be one of the most stable types of phages however, a number of studies suggest considerable variation in phage stability exposed to identical stress conditions [72]. We have shown that phage CDKM9 may be formulated with Eudragit® in a 0.2 M salt solution. Merabishvili et al. [34] showed that an S. aureus phage (from the *Myoviridae* family) formulated in ~2 mM salt solution may be stored under refrigerated conditions for up to 3 months without loss of phage titre. Generally, BHI broth is the storage medium of choice for phage stocks; it is known to keep phages stable whilst stored over long periods of time [78]. Although phage CDKM9 may be formulated with Eudragit® in BHI, we decided to avoid working with a complex broth medium since both saline and broth showed similar phage viability results.

A number of previous studies have used alginate as the main encapsulating agent either on its own [37], [63], [64] or in combination with whey protein [32], [60], [61] or chitosan [31], [33]. Phages encapsulated in crosslinked pure alginate microparticles were found to be susceptible to acid damage following exposure to simulated gastric fluid [31], [37], [62]. Alginate hydrogel pores tend to be in the 5–200 nm range depending on the degree of crosslinking [79]. The porosity of the alginate gel microparticles affects phage susceptibility to acid damage due to diffusion of acid. A number of studies have added CaCO_3_ within the microparticles to protect phage from acid damage [37], [63]. Other studies have shown that by combining alginate with chitosan [31], [80] or whey protein [32], [60], [61], better acid protection may be achieved. We have shown that by combining the pH responsive character of Eudragit® with alginate, phage CDKM9 was significantly protected from exposure to simulated gastric fluid at pH 2 and could thereafter be readily released upon exposure to pH 7. Furthermore, the microparticles were stable upon storage under refrigerated conditions for up to 4 weeks. The combination of small particle size and pH responsive character of the encapsulating polymer resulted in rapid release of phage within the first hour upon exposure to pH 7 whereas release was negligible at pH 6. Previous studies on phage encapsulated in large microparticles (~ 1mm) have reported slower sustained release kinetics for alginate encapsulated phage [31], [33]. Colom et al. [63] reported faster release from small alginate microparticles containing CaCO_3_ as antacid (mean size ~ 100 μm). However, exposure to simulated gastric fluid (pH 2.8 for 60 min) resulted in between 2 log and 3 log reduction in *Salmonella* phage titre suggesting that even with the addition of CaCO_3_, phage were highly susceptible to SGF. Particle size was found to be an important factor influencing phage protection from SGF for acid permeable beads [61]. Alginate-whey protein microparticles having the same composition but different mean sizes showed larger particles protected phage better compared with smaller microparticles [61]. The diffusion path length of acid is increased with particle size thereby affording protection to phage further away from the bead surface. We have shown that encapsulation of phage in Eudragit®/alginate microparticles (mean size ~ 100 μm) may permit rapid burst release of phage cargo without significant acid damage. In future studies, it may be possible to tailor phage release kinetics through controlling the microparticle size and the shell composition (e.g. Eudragit to alginate ratio); this work is currently underway in our laboratory.

A number of *in vivo* animal studies have shown dose dependent phage therapy outcomes, with high doses of phage resulting in better clinical results [81–84]. Accurate control over the phage dose delivered at the site of infection and the timing of the delivery are important considerations [14]. Careful formulation and microfluidic loading of phage (resulting in uniform particle size) would facilitate control over the phage release dynamics and accurate delivery of the phage dosage. This has so far received little attention in the published literature. We have demonstrated that even after exposure of phage encapsulated microparticles to simulated gastric fluid at pH 2 for 3 hours, the released phage dose corresponds to ~ 1 x 10^7^ PFU g^-1^ of phage microparticles. The encapsulated dose was ~ 2 x 10^8^ PFU g^-1^ of phage microparticles. One gram of microparticles would therefore deliver a ~10^7^ PFU dose which is similar to therapeutic doses given in a number of animal studies [28], [85–89]. Nale et al. [28] in their hamster *C*. *difficile* infection model delivered an unformulated phage suspension at a dose of ~ 10^8^ PFU/hamster. The actual dose delivered to the site of infection would have been considerably lower. Failure of the recently concluded phage trial for the treatment of *E coli* diarrhoea in children was attributed to the lack of clinical host range coverage of the employed *E*.*coli* phages to the disease-causing bacteria (faecal phage titres were not higher in patients harbouring a phage sensitive E. coli colony in the stool; around 50% of all patients). A drop in phage titre due to phage exposure to stomach acidity may also have been a factor in the consequent lack of *in situ* phage amplification [14]. Stanford et al. [90] previously used Eudragit® S100, for phage encapsulation and enteric delivery targeting *E*. *coli* infection in cattle. A spray dryer was used to encapsulate phages into a dry powder, which was administered either as a bolus or added to the feed directly. The method was not optimised to protect the phage from the stomach acidity. Some protection of phages from stomach acidic pH was seen with phage replicating *in vivo* and a lower *E*. *coli* faecal shedding rate was observed in phage treated cattle. We have demonstrated much improved acid protection of phage encapsulated in highly uniform Eudragit®/alginate microparticles; a significant step forward compared with previously published work.

Delivery systems and their formulation require consideration of the bioavailabity of the active agent at the site of infection. The size of microparticles may play an important role by ensuring it is minimally affected by the conditions of the diseased state. The small intestine and the colon are connected via a junction known as the ileum; while *C*. *difficile* infection has been reported to occur and cause disease in the small intestine [91], in most patients the bacterium mainly affects the colon. Nevertheless, release of phage in the small intestine as microparticles transit towards the colon may prove beneficial in eradicating CDI for a wide range of patients. Other considerations for colon targeted delivery of active agents include the changes in transit times during infection. Increased loss of fluid from the colon due to symptoms such as diarrhoea result in observed shortened mean transit times. Dilution of phage due to the high fluid environment may be a particular challenge. Larger particles (~ mm size range) may be more prone to the influence of short transit times. Smaller particles may be predisposed to non-specific mucoadhesion [92], [93] which may aid in phage retention and sustained release over a signficant time period. Colom et al. [63] showed better phage retention (for animals treated with encapsulated phage versus those treated with free phage) in the caecum of chickens and a significant reduction in *Salmonella* colonisation, attributing these results to the mucoadhesiveness of the small alginate microparticles (~ 100 μm) used for encapsulating phage. Wittaya-areekul et al., [94] showed differences in mucoadhesion properties of chitosan coated alginate microparticles using an *in vitro* assay using porcine gut tissue samples.

Gastrointestinal infections can affect different parts of the gut. In future different phage fomulations may need to be developed targeting phage release in the appropriate intestinal region perhaps using more specific infection related triggers e.g. enzymes or toxins released by the bacterium causing the infection [95].

A limitation of phages for therapeutic use (*C*. *difficile* phages such as CDKM9 are no exception) is their narrow host range. Most phages only infect a small number of prevalent strains, thereby necessitating the use of phage cocktails to provide adequate therapeutic coverage. Phages incorporated in the cocktail may require tailored formulations to ensure adequate stability [96].

Microfluidic systems such as the one described in the present work have the potential to enable precise control of the particle size and loading of the phages within the microparticles. Here, we have demonstrated for the first time, proof-of-concept data in support of this platform technology for the production of encapsulated phage in a pH responsive polymer suitable for targeted intestinal phage delivery. An eight week study on the use of Eudragit® coated mesalazine tablets in patients with mild to moderately active ulcerative colitis [97] showed that the tablets were well tolerated suggesting Eudragit® based microencapsulation of phages may be safe for use in humans.

## Conclusions

Highly uniform small microparticles (mean size ~ 100 μm) of Eudragit® S100 or Eudragit® S100/alginate were generated using a flow-focussing glass capillary device. A novel mechanism of microparticle formation was demonstrated using acidified medium-chain triglycerides in a water-in-oil emulsion system. Solid core-shell microparticles were produced with a low polydispersity index enabling control over phage loading and their subsequent pH triggered release. Encapsulation of carboxyfluorescein in Eudragit® S100 particles was visualised using confocal laser scanning microscopy and examined under different conditions elucidating the effect of different formulation and microfluidic experimental parameters on particle size. Phage encapsulation and subsequent release kinetics revealed that microparticles prepared using Eudragit® S100 formulations possess pH responsive characteristics with phage release triggered in an intestinal pH range suitable for therapeutic purposes. The encapsulated phages were shown to be significantly protected upon prolonged exposure to an acidic environment without the addition of an antacid in the formulation. We have also demonstrate the storage stability of the encapsulated phage under refrigerated conditions over a 4 week storage period. Future work will investigate the efficacy of the encapsulated phages by controlling phage loading and release rates on bacterial inactivation in suitable *in vitro* chemostat type systems as well as in tissue culture models and long term in relevant animal infection models.

## Supporting information

S1 Fig Release of encapsulated phage from alginate microparticles. Phage release kinetics from alginate microparticles exposed to simulated intestinal fluid (SIF) at pH 6 and pH 7 and after exposure to simulated gastric fluid (SGF) ay pH 2 (exposure for 3 hours to SGF) followed by dissolution of microparticles in SIF at pH 7. 0 hours time point denotes exposure time between 0–10 min. Error bars indicate 95% confidence intervals for means.
(TIF)
