# Supplementary figures and images for: Microencapsulation of Clostridium difficile specific bacteriophages using microfluidic glass capillary devices for colon delivery using pH triggered release
Source: PLoS One. 2017 Oct 12;12(10):e0186239. doi: 10.1371/journal.pone.0186239 (PMC5638336; doi:10.1371/journal.pone.0186239)

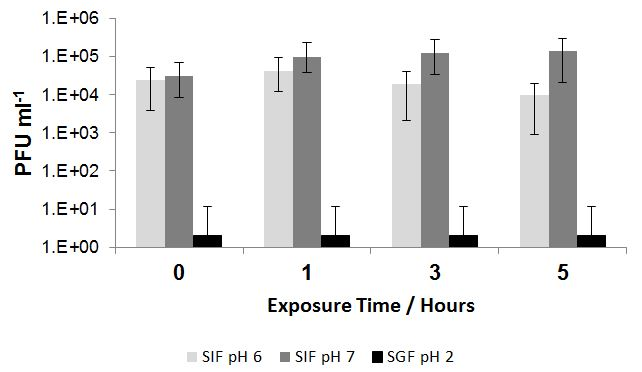

Supplement: S1 Fig — Phage release kinetics from alginate microparticles exposed to simulated intestinal fluid (SIF) at pH 6 and pH 7 and after exposure to simulated gastric fluid (SGF) ay pH 2 (exposure for 3 hours to SGF) followed by dissolution of microparticles in SIF at pH 7. 0 hours time point denotes exposure time between 0–10 min. Error bars indicate 95% confidence intervals for means. (TIF) [file pone.0186239.s001.tif]
